# Molecular Evidence for *Anaplasma phagocytophilum* in Israel

**DOI:** 10.3201/eid1309.070455

**Published:** 2007-09

**Authors:** Avi Keysary, Robert F. Massung, Moshe Inbar, Arian D. Wallach, Uri Shanas, Kosta Y. Mumcuoglu, Trevor Waner

**Affiliations:** * Israel Institute for Biological Research, Ness Ziona, Israel; † Centers for Disease Control and Prevention, Atlanta, Georgia, USA; ‡ University of Haifa, Haifa, Israel; § Hebrew University–Hadassah Medical School, Jerusalem, Israel

**Keywords:** Anaplasma phagocytophilum, ticks, Israel, dispatch

## Abstract

Sequences from the *Anaplasma phagocytophilum* 16S rRNA gene were detected in 5 ticks representing 3 species (*Hyalomma marginatum, Rhipicephalus turanicus,* and *Boophilus kohlsi*) collected from roe deer (*Capreolus capreolus*) in Mount Carmel, Israel. The sequences were all identical to those of Ap-variant 1 strain.

*Anaplasma phagocytophilum* is the causative agent of granulocytic anaplasmosis (ehrlichiosis) in humans, horses, sheep, cattle, dogs, and cats (*1*). Although serologic evidence for the presence of *A. phagocytophilum* in humans (*2*), jackals (*3*), and domestic dogs (*4*) has been available in Israel since 1999, no direct verification has been presented to confirm its occurrence. In this study, we present molecular evidence for the occurrence of *A. phagocytophilum* in ticks in Israel collected from roe deer.

Ticks were collected from 4 female roe deer (*Capreolus capreolus*) between 2004 and 2005. The deer were part of a reintroduction program initiated in Israel, with deer imported since 1991 from France, Italy, and Hungary and brought to the Hai-Bar Carmel breeding facility on Mount Carmel (*5*). The collected ticks were kept in a 70% ethanol solution for identification and DNA extraction.

Extraction of DNA was performed by using the QIAamp Minikit Catalogue no. 51304 (QIAGEN Inc., Valencia, CA, USA). The DNA extract from each tick was tested for *A. phagocytophilum* by using a nested PCR assay that amplified a 456-bp portion of the 5′ region of the 16S rRNA gene as previously described (*6*). Each positive PCR product was subjected to DNA sequencing with fluorescent-labeled dideoxynucleotide technology (BigDye Terminator Cycle Sequencing Ready Reaction Kit; Applied Biosystems, Foster City, CA, USA). Sequencing reaction products were separated, and data were collected by using an ABI 3100 Genetic Analyzer automated DNA sequencer (Applied Biosystems).

Seventy ticks were collected from roe deer. DNA extracted from 5 (7.1%) of the 70 ticks produced products when primers specific to the16S rRNA gene of *A. phagocytophilum* were used (Table). DNA sequences from 16S rRNA of *A. phagocytophilum* from the ticks showed a high degree of homology with those reported in the GenBank database. All sequences examined from the ticks were identical. They all differed by 2 bp from the sequence of the human agent (Ap-ha) (GenBank accession no. U02521) but were identical to the variant strain referred to as AP-variant 1 (GenBank accession no. AY193887) (*6*).

**Table:** PCR positivity to *Anaplasma phagocytophilum* in ticks collected from roe deer (*Capreolus capreolus*), Mount Carmel, Israel

Tick species	No. ticks tested	Ticks with *A. phagocytophilum* DNA
*Rhipicephalus turanicus*		
Females	25	2
Males	16	1
*R. sanguineus*	1	0
*Hyalomma marginatum*		
Females	4	0
Males	10	1
*Boophilus kohlsi*		
Males	1	0
Nymphs	13	1

*Rhipicephalus* *sanguineus* and *R. turanicus* ticks are common in Israel and found on a large variety of domestic and wild animals (*7*). *Hyalomma marginatum* ticks have a worldwide distribution and have been documented on mountain gazelles and Nubian ibexes in Israel (*8*); *Boophilus kohlsi* has been documented on sheep and goats in Jordan (*9*). In a study conducted in Spain, *A. phagocytophilum* was found in *Dermacenter marginatus, Ixodes ricinus, R. bursa,* and *Hemophysalis punctata* (*10*). Santos-Silva et al. were not able to demonstrate the presence of *A. phagocytophilum* in *H. marginatum* or *R. turanicus* in Portugal (*11*). Existing evidence cannot determine whether these ticks could act as vectors of *A. phagocytophilum* or were merely infected during a blood meal from an infected roe deer.

The presence of *A. phagocytophilum* in roe deer has been demonstrated in Slovakia (*12*), Germany (*13*), the Czech Republic, and Austria (*14*). These data indicate that roe deer may act as reservoirs for *A. phagocytophilum* in Israel. The primary reservoir for the Ap-variant 1 strain in the United States has been reported to be white-tailed deer (*15*). Although this strain has never been associated with a human infection, additional studies are needed to define its host range and pathogen potential. Our study presents molecular evidence of the presence of *A. phagocytophilum* in ticks in Israel and could have important implications for both medical and veterinary healthcare providers.
